# Association of the Usability of Electronic Health Records With Cognitive Workload and Performance Levels Among Physicians

**DOI:** 10.1001/jamanetworkopen.2019.1709

**Published:** 2019-04-05

**Authors:** Lukasz M. Mazur, Prithima R. Mosaly, Carlton Moore, Lawrence Marks

**Affiliations:** 1 School of Information and Library Science, University of North Carolina at Chapel Hill, Chapel Hill; 2 Carolina Health Informatics Program, University of North Carolina at Chapel Hill, Chapel Hill; 3 Division of Healthcare Engineering, Department of Radiation Oncology, University of North Carolina at Chapel Hill, Chapel Hill; 4 Division of General Medicine, University of North Carolina at Chapel Hill, Chapel Hill

## Abstract

**Importance:**

Current electronic health record (EHR) user interfaces are suboptimally designed and may be associated with excess cognitive workload and poor performance.

**Objective:**

To assess the association between the usability of an EHR system for the management of abnormal test results and physicians’ cognitive workload and performance levels.

**Design, Setting, and Participants:**

This quality improvement study was conducted in a simulated EHR environment. From April 1, 2016, to December 23, 2016, residents and fellows from a large academic institution were enrolled and allocated to use either a baseline EHR (n = 20) or an enhanced EHR (n = 18). Data analyses were conducted from January 9, 2017, to March 30, 2018.

**Interventions:**

The EHR with enhanced usability segregated in a dedicated folder previously identified critical test results for patients who did not appear for a scheduled follow-up evaluation and provided policy-based decision support instructions for next steps. The baseline EHR displayed all patients with abnormal or critical test results in a general folder and provided no decision support instructions for next steps.

**Main Outcomes and Measures:**

Cognitive workload was quantified subjectively using NASA–Task Load Index and physiologically using blink rates. Performance was quantified according to the percentage of appropriately managed abnormal test results.

**Results:**

Of the 38 participants, 25 (66%) were female. The 20 participants allocated to the baseline EHR compared with the 18 allocated to the enhanced EHR demonstrated statistically significantly higher cognitive workload as quantified by blink rate (mean [SD] blinks per minute, 16 [9] vs 24 [7]; blink rate, –8 [95% CI, –13 to –2]; *P* = .01). The baseline group showed statistically significantly poorer performance compared with the enhanced group who appropriately managed 16% more abnormal test results (mean [SD] performance, 68% [19%] vs 98% [18%]; performance rate, –30% [95% CI, –40% to –20%]; *P* < .001).

**Conclusions and Relevance:**

Relatively basic usability enhancements to the EHR system appear to be associated with better physician cognitive workload and performance; this finding suggests that next-generation systems should strip away non–value-added EHR interactions, which may help physicians eliminate the need to develop their own suboptimal workflows.

## Key Points

**Question:**

Is enhanced usability of an electronic health record system associated with physician cognitive workload and performance?

**Findings:**

In this quality improvement study, physicians allocated to perform tasks in an electronic health record system with enhancement demonstrated statistically significantly lower cognitive workload; those who used a system with enhanced longitudinal tracking appropriately managed statistically significantly more abnormal test results compared with physicians allocated to use the baseline electronic health record.

**Meaning:**

Usability improvements in electronic health records appear to be associated with improved cognitive workload and performance levels among clinicians; this finding suggests that next-generation systems should strip away non–value-added interactions.

## Teaser

This quality improvement study analyzes the perceived and physiological workload, performance, and fatigue levels of residents and fellows as they manage abnormal or critical test results using an electronic health record (EHR) system.

## Introduction

The usability of electronic health records (EHRs) continues to be a major concern.^1,2,3^ Usability challenges include suboptimal design of interfaces that have confusing layouts and contain either too much or too little relevant information as well as workflows and alerts that are burdensome. Suboptimal usability has been associated with clinician burnout and patient safety events, and improving the usability of EHRs is an ongoing need.^4,5^

A long-standing challenge for the US health care system has been to acknowledge and appropriately manage abnormal test results and associated missed or delayed diagnoses.^6,7,8,9,10,11^ The unintended consequences of these shortcomings include missed and delayed cancer diagnoses and associated negative clinical outcomes (eg, 28% of women did not receive timely follow-up for abnormal Papanicolaou test results^8^; 28% of women requiring immediate or short-term follow-up for abnormal mammograms did not receive timely follow-up care^9^). Even in the EHR environment, with alerts and reminders in place, physicians continue to often inappropriately manage abnormal test results.^12,13,14,15,16,17,18,19,20,21^ Some key remaining barriers to effective management of test results are suboptimal usability of existing EHR interfaces and the high volume of abnormal test result alerts, especially less-critical alerts that produce clutter and distract from the important ones.^22,23^ In addition, few organizations have explicit policies and decision support systems in their EHR systems for managing abnormal test results, and many physicians have developed processes on their own.^24,25,26^ These issues are among the ongoing reasons to improve the usability of the EHR-based interfaces for the evaluation and management of abnormal test results.

We present the results of a quality improvement study to assess a relatively basic intervention to enhance the usability of an EHR system for the management of abnormal test results. We hypothesized that improvements in EHR usability would be associated with improvements in cognitive workload and performance among physicians.

## Methods

### Participants

This research was reviewed and approved by the institutional review board committee of the University of North Carolina at Chapel Hill. Written informed consent was obtained from all participants. The study was performed and reported according to the Standards for Quality Improvement Reporting Excellence (SQUIRE) guideline.^27^

Invitations to participate in the study were sent to all residents and fellows in the school of medicine at a large academic institution, clearly stating the need for experience with using the Epic EHR software (Epic Systems Corporation) in reviewing test results to undergo the study’s simulated scenarios. A $100 gift card was offered as an incentive for participation. Potential participants were given an opportunity to review and sign a consent document, which included information on study purpose, goals, procedures, and risks and rewards as well as the voluntary nature of participation and the confidentiality of data. Recruited individuals had the right to discontinue participation at any time. Forty individuals were recruited to participate, 2 of whom were excluded (eg, numerous cancellations), leaving 38 evaluable participants (Table 1).

**Table 1.** Composition of Participants

Variable	No. (%)					
	Internal Medicine Specialty	Family Medicine Specialty	Pediatrics Specialty	Surgery Specialty	Other Specialty	Total
All patients	14 (37)	4 (11)	9 (24)	5 (13)	6 (16)	38
Baseline EHR	9 (45)	3 (15)	3 (15)	2 (10)	3 (15)	20
Enhanced EHR	5 (28)	1 (6)	6 (33)	3 (17)	3 (17)	18
Postgraduate year						
1	4 (40)	1 (10)	3 (30)	1 (10)	1 (10)	10
2	2 (25)	1 (13)	2 (25)	2 (25)	1 (13)	8
3	5 (45)	1 (9)	4 (36)	0	1 (9)	11
4	3 (43)	1 (14)	0	1 (14)	2 (29)	7
5	0	0	0	1 (50)	1 (50)	2
Sex						
Male	5 (38)	2 (15)	2 (15)	1 (8)	3 (23)	13
Female	9 (43)	2 (4)	7 (28)	4 (16)	3 (12)	25

Abbreviation: EHR, electronic health record.

### Study Design 

From April 1, 2016, to December 23, 2016, 38 participants were enrolled and prospectively and blindly allocated to a simulated EHR environment: 20 were assigned to use a baseline EHR (without changes to the interface), and 18 were assigned to use enhanced EHRs (with changes intended to enhance longitudinal tracking of abnormal test results in the system) (Figure). Abnormalities requiring an action included new abnormal test results and previously identified abnormal test results for patients who did not show up (without cancellation) for their scheduled appointment in which the findings would be addressed. The new abnormal test results included a critically abnormal mammogram (BI-RADS 4 and 5) and Papanicolaou test result with high-grade squamous intraepithelial lesion as well as noncritical results for rapid influenza test, streptococcal culture complete blood cell count, basic metabolic panel, and lipid profile, among others. The previously identified critical test results that required follow-up included abnormal mammogram (BI-RADS 4 and 5), Papanicolaou test result with high-grade squamous intraepithelial lesion, chest radiograph with 2 × 2-cm lesion in the left upper lobe, pulmonary function test result consistent with severe restrictive lung disease, and pathologic examination with biopsy finding of ascending colon consistent with adenocarcinoma.

**Figure.**
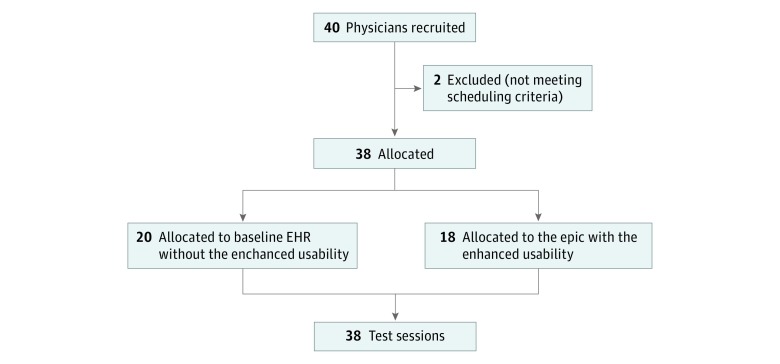
Study Design EHR indicates electronic health record.

The simulated scenarios were iteratively developed and tested by an experienced physician and human factors engineer (C.M. and L.M.) in collaboration with an Epic software developer from the participating institution. The process included functionality and usability testing and took approximately 12 weeks to complete. The experimental design was based on previous findings that attending physicians use the EHR to manage approximately 57 test results per day over multiple interactions.^22,23^ Given that residents often manage a lower volume of patients, the present study was designed such that participants were asked to review a total of 35 test results, including 8 or 16 abnormal test results evenly distributed between study groups, in 1 test session. By organizational policies and procedures, participants were expected to review all results, acknowledge and follow-up on abnormal test results, and follow-up on patients with a no-show status (without cancellation) for their scheduled appointment aimed at addressing their previously identified abnormal test result. The patient data in the simulation included full medical records, such as other clinicians' notes, previous tests, and other visits or subspecialist coverage.

### Intervention

The baseline EHR (without enhanced interface usability), currently used at the study institution, displayed all new abnormal test results and previously identified critical test results for patients with a no-show status (did not show up for or cancelled their follow-up appointment) in a general folder called Results and had basic sorting capabilities. For example, it moved all abnormal test results with automatically flagged alerts to the top of the in-basket queue; flagged alerts were available only for test results with discrete values. Thus, critical test results for mammography, Papanicolaou test, chest radiograph, pulmonary function test, and pathologic examination were not flagged or sortable in the baseline EHR. The baseline EHR included patient status (eg, completed the follow-up appointment, no show), however, that information needed to be accessed by clicking on the visit or patient information tab located on available prebuilt views within each highlighted result.

The enhanced EHR (with enhanced interface usability) automatically sorted all previously identified critical test results for patients with a no-show status in a dedicated folder called All Reminders. It also clearly displayed information regarding patient status and policy-based decision support instructions for next steps (eg, “No show to follow-up appointment. Reschedule appointment in Breast Clinic”).

The intervention was developed according to the classic theory of attention.^28^ This theory indicates that cognitive workload varies continuously during the course of performing a task and that the changes of cognitive workload may be attributed to the adaptive interaction strategies of the operator exposed to task demands (eg, baseline or enhanced usability).

### Main Outcomes and Measures

#### Perceived Workload

The NASA–Task Load Index (NASA-TLX) is a widely applied and valid tool used to measure workload,^29,30,31,32,33,34^ including the following 6 dimensions: (1) mental demand (How much mental and perceptual activity was required? Was the task easy or demanding, simple or complex?); (2) physical demand (How much physical activity was required? Was the task easy or demanding, slack or strenuous?); (3) temporal demand (How much time pressure did you feel with regard to the pace at which the tasks or task elements occurred? Was the pace slow or rapid?); (4) overall performance (How successful were you in performing the task? How satisfied were you with your performance?); (5) frustration level (How irritated, stressed, and annoyed [compared with content, relaxed, and complacent] did you feel during the task?); and (6) effort (How hard did you have to work, mentally and physically, to accomplish your level of performance?).

At the end of the test session, each participant performed 15 separate pairwise comparisons of the 6 dimensions (mental demand, physical demand, temporal demand, overall performance, frustration level, and effort) to determine the relevance (and hence weight) of a dimension for a given session for a participant. Next, participants marked a workload score between low (corresponding to 0) to high (corresponding to 100), separated by 5-point marks on the tool, for each dimension for each session. The composite NASA-TLX score for each session was obtained by multiplying the dimension weight with the corresponding dimension score, summing across all dimensions, and dividing by 15.

#### Physiological Workload

Using eye-tracking technology (Tobii X2-60 screen mount eye tracker; Tobii), we quantified physiological workload with validated methods based on changes in blink rate.^35,36^ Eye closures ranging between 100 milliseconds to 400 milliseconds were coded as a blink. The validity (actual blink or loss of data) was later confirmed by visual inspection by the expert researcher on our team (P.R.M.) who specializes in physiological measures of cognitive workload. Decreased blink rate has been found to occur in EHR-based tasks requiring more cognitive workload.^37^ The fundamental idea is that blink rate slows down under visual task demands that require more focused attention and working memory load, but this association might vary with the type of visual task demands.^38,39,40^ For each participant, the time-weighted mean blink rate measured during the participant’s review of all abnormal test results was calculated and then considered for data analysis.

#### Performance

For each participant, performance was quantified as the percentage of (new or previously identified) abnormal test results that were appropriately acted on (with possible scores ranging from 0%-100%). Appropriate action on abnormal test result was defined as the study participant ordering (compared with not ordering) a referral for further diagnostic testing (eg, breast biopsy for mass identified on an abnormal mammogram) to a subspecialty clinic (eg, breast clinic). In addition, per the policy and procedures of the institution in which the study took place, if patients missed their appointment for follow-up on critical test results, the participants were expected to contact (compared with not contact) schedulers to reschedule follow-up care. We also quantified the total amount of time that participants took to complete each simulated scenario.

### Secondary Outcome and Measure

Fatigue can affect perceived and physiological workload and performance and thus can confound study results.^41,42,43^ Because of the possible confounding association of fatigue, participants were asked to evaluate their own state of fatigue immediately before each simulated session using the fatigue portion of the Crew Status Survey.^44^ The fatigue assessment scale included these levels: 1 (fully alert, wide awake, or extremely peppy), 2 (very lively, or responsive but not at peak), 3 (okay, or somewhat fresh), 4 (a little tired, or less than fresh), 5 (moderately tired, or let down), 6 (extremely tired, or very difficult to concentrate), and 7 (completely exhausted, unable to function effectively, or ready to drop). The Crew Status Survey has been tested in real and simulated environments and has been found to be both reliable and able to discriminate between fatigue levels.^44,45^

### Statistical Analysis

On the basis of the anticipated rate of appropriately identified abnormal test results in the literature^12,13,14,15,16,17,18,19,20,21^ and the anticipated magnitude of the association of the enhanced EHR, we required a sample size of 30 participants, each reviewing 35 test results, to achieve 80% power to detect a statistically significant difference in cognitive workload and performance. Specifically, we performed sample size calculations at α = .05, assuming that we could detect a mean (SD) difference of 10 (10) in NASA-TLX scores, a mean (SD) difference of 5 (10) in blink rate, and a mean (SD) difference of 10% (15%) in performance.

Before data analyses, we completed tests for normality using the Shapiro-Wilk test and equal variance using the Bartlett test for all study variables (cognitive workload, performance, and fatigue). Results indicated that all assumptions to perform parametric data analysis were satisfied (normality: all *P* > .05; equal variance: all *P* > .05).

We conducted a 2-sample *t* test to assess the association of enhanced usability of the EHR interface to manage abnormal test results with physician cognitive workload and performance. All data analyses were conducted from January 9, 2017, to March 30, 2018, using JMP 13 Pro software (SAS Institute Inc). Statistical significance level was set at 2-sided *P* = .05, with no missing data to report.

## Results

Of the 852 eligible residents and fellows, 38 (5%) participated. Twenty-five participants (66%) were female and 13 (34%) were male. Thirty-six (95%) were residents and 2 (5%) were fellows (Table 1). Descriptive statistics of cognitive workload and performance are provided in Table 2.

**Table 2.** Perceived and Physiological Quantification of Cognitive Workload and Performance

Workload and Performance	Mean (SD)		
	Baseline EHR	Enhanced EHR	*P* Value
Perceived workload			
NASA-TLX score^a^	53 (14)	49 (16)	.41
Mental demand (mean weight: 3.67)	66 (15)	53 (19)	.02
Physical demand (mean weight: 0.19)	18 (10)	15 (12)	>.05
Temporal demand (mean weight: 2.83)	49 (24)	49 (22)	>.05
Performance demand (mean weight: 3.56)	37 (15)	39 (15)	>.05
Effort (mean weight: 2.67)	59 (21)	54 (17)	>.05
Frustration (mean weight: 2.08)	45 (28)	47 (21)	>.05
Cognitive workload			
Blink rate, blinks/min, No.	16 (9)	24 (7)	.01
Performance, No. appropriately managed/No. of failure opportunities (%)^b^			
Overall	152/210 (68)	170/189 (89)	<.001
New abnormal test results	118/120 (98)	108/108 (100)	>.05
Previously identified critical test results for patients with no-show status	34/90 (37)	62/81 (77)	<.001
Time to complete scenario, s	238 (83)	236 (77)	>.05

Abbreviations: EHR, electronic health record; NASA-TLX, NASA–Task Load Index.

^a^ The NASA-TLX tool was used to measure workload,^29,30,31,32,33,34^ including 6 dimensions. Score range: 0 (low) to 100 (high).

^b^ Performance was the percentage of (new or previously identified) abnormal test results that were appropriately acted on. Possible scores ranged from 0% to 100%.

### Perceived and Physiological Workload

No statistically significant difference was noted in perceived workload between the baseline EHR and enhanced EHR groups (mean [SD] NASA-TLX score, 53 [14] vs 49 [16]; composite score, 4 [95% CI, –5 to 13]; *P* = .41). A statistically significantly higher cognitive workload as shown by the lower mean blink rate was found in the baseline EHR group compared with the enhanced EHR group (mean [SD] blinks per minute, 16 [9] vs 24 [7]; blink rate, –8 [95% CI, –13 to –2]; *P* = .01).

### Performance

A statistically significantly poorer performance was found in the baseline EHR group compared with the enhanced EHR group (mean [SD] performance, 68% [19%] vs 98% [18%]; performance rate, –30% [95% CI, –40% to –20%]; *P* < .001). The difference was mostly attributable to review of patients with a no-show status for a follow-up appointment (Table 2). No difference between the baseline and enhanced EHR groups was noted in time to complete simulated scenarios (mean [SD] time in seconds, 238 [83] vs 236 [77]; time to complete, 2 seconds [95% CI, –49 to 52]; *P* > .05). No statistically significant difference was noted in fatigue levels between baseline and enhanced EHR groups (mean [SD] fatigue level, 2.7 [1.4] vs 2.8 [0.9]; fatigue level, –0.1 [95% CI, –0.8 to 0.7]; *P* = .84).

The rate of appropriately managing previously identified critical test results of patients with a no-show status in the baseline EHR was 37% (34 of 90 failure opportunities) compared with 77% (62 of 81 failure opportunities) in the enhanced EHR. The rate of appropriately acknowledging new abnormal test results in the baseline EHR group was 98% (118 of 120 failure opportunities; 2 participants did not acknowledge a critical Papanicolaou test result) compared with 100% (108 of 108 failure opportunities) in the enhanced EHR group.

## Discussion

Participants in the enhanced EHR group indicated physiologically lower cognitive workload and improved clinical performance. The magnitude of the association of EHR usability with performance we found in the present study was modest, although many such improvements tend to have substantial value in the aggregate. Thus, meaningful usability changes can and should be implemented within EHRs to improve physicians’ cognitive workload and performance. To our knowledge, this research is the first prospective quality improvement study of the association of EHR usability enhancements with both physiological measure of cognitive workload and performance during physicians’ interactions with the test results management system in the EHR.

The enhanced EHR was more likely to result in participants reaching out to patients and schedulers to ensure appropriate follow-up. Physicians who used the baseline EHR were more likely to treat the EHR (not treat the patient) by duplicating the referral, rather than to reach out to patients and schedulers to find out the issues behind the no-show. In the poststudy conversations with participants, most indicated a lack of awareness about policies and procedures for managing patients with a no-show status and justified their duplication of orders as safer medial practice. This result seems to be in line with findings from real clinical settings, suggesting that few organizations have explicit policies and procedures for managing test results and most physicians developed processes on their own.^25,26^

The result from the baseline EHR group is in line with findings from real clinical settings that indicated physicians did not acknowledge abnormal test results in approximately 4% of cases.^19,20^ The optimal performance in the enhanced EHR group is encouraging.

No significant difference was noted in the time to complete simulated scenarios and perceived workload between baseline and enhanced EHR groups, as quantified by the global NASA-TLX or by each dimension, while trending toward lower scores (Table 2). The time to complete simulated scenarios and NASA-TLX scores was elevated in the participants in the enhanced EHR group possibly because it was their first time interacting with this enhanced usability.

Overall, past and present research suggests that challenges remain in ensuring the appropriate management of abnormal test results. According to a study, 55% of clinicians believe that EHR systems do not have convenient usability for longitudinal tracking of and follow-up on abnormal test results, 54% do not receive adequate training on system functionality and usability, and 86% stay after hours or come in on the weekends to address notifications.^46^

We propose several interventions based on our findings to improve the proper management of abnormal test results. First, use the existing capabilities and usability features of the EHR interfaces to improve physicians’ cognitive workload and performance. Similar recommendations were proposed by other researchers.^3,5,17,18,19,20,21,46,47,48^ For example, the critical test results for patients with a no-show status should be flagged (ie, clearly visible to the clinician) indefinitely until properly acted on in accordance with explicit organizational policies and procedures. Second, develop explicit policies and procedures regarding the management of test results within EHRs, and implement them throughout the organization, rather than having clinicians develop their own approaches.^25,26,49^ For example, Anthony et al^49^ studied the implementation of a critical test results policy for radiology that defined critical results; categorized results by urgency and assigned appropriate timelines for communication; and defined escalation processes, modes of communication, and documentation processes. Measures were taken for 4 years from February 2006 to January 2010, and the percentage of reports adhering to the policies increased from 29% to 90%.^49^ Third, given that the work is being done in an electronic environment, seize the opportunities to use innovative simulation-based training sessions to address the challenges of managing test results within an EHR ecosystem.^50,51,52,53,54^ Fourth, establish a regular audit and feedback system to regularly give physicians information on their performance on managing abnormal test results.^55,56,57^

This study focused on a particular challenge (ie, the management of abnormal test results), but many other interfaces and workflows within EHRs can be similarly enhanced to improve cognitive workload and performance. For example, there is a need to improve reconciliation and management of medications, orders, and ancillary services. The next generation of EHRs should optimize usability by stripping away non–value-added EHR interactions, which may help eliminate the need for physicians to develop suboptimal workflows of their own.

### Limitations

This study has several limitations, and thus caution should be exercised in generalizing the findings. First, the results are based on 1 experiment with 38 residents and fellows from a teaching hospital artificially performing a discrete set of scenarios. Larger studies could consider possible confounding factors (eg, specialty, training levels, years of EHR use, attendings or residents) and more accurately quantify the association of usability with cognitive workload and performance. Second, performing the scenarios in the simulated environment, in which the participants knew that their work was going to be assessed, may have affected participants’ performance (eg, more or less attentiveness and vigilance as perceived by being assessed or by the possibility of real harm to the patient). To minimize this outcome, all participants were given a chance to discontinue their participation at any time, but participant-specific findings would remain confidential. None of the participants discontinued participation in the study, although 2 participants were excluded from the study as they were not able to meet the scheduling criteria. Third, we acknowledge that the cognitive workload and performance scores were likely affected by the setting (eg, simulation laboratory and EHR) and thus might not reflect the actual cognitive workload and performance in real clinical settings. A laboratory setting cannot totally simulate the real clinical environment, and some activities cannot be easily reproduced (eg, looking up additional information about the patient using an alternative software, calling a nurse with a question about a particular patient, or a radiologist or laboratory technician calling physicians and verbally telling them about abnormal images). We also recognize that the enhanced usability was not optimal as it was designed and implemented within the existing capabilities of the EHR environment used for training purposes.

Fourth, the intervention might have manipulated both the ease of access to information through a reorganized display and learning because it provided a guide to action by clearly showing information on patient status and policy-based decision support instructions for next steps. Future research could more accurately quantify the association of usability and learning with cognitive workload and performance. Nevertheless, the intervention provided the necessary basis to conduct this study. All participants were informed about the limitations of the laboratory environment before the study began.

## Conclusions

Relatively basic usability enhancements to EHR systems appear to be associated with improving physician management of abnormal test results while reducing cognitive workload. The findings from this study support the proactive evaluation of other similar usability enhancements that can be applied to other interfaces within EHRs.
